# *KCNV2* retinopathy: clinical features, molecular genetics and directions for future therapy

**DOI:** 10.1080/13816810.2020.1766087

**Published:** 2020-05-22

**Authors:** Thales A. C. De Guimaraes, Michalis Georgiou, Anthony G. Robson, Michel Michaelides

**Affiliations:** a UCL Institute of Ophthalmology, University College London, London, UK; b Moorfields Eye Hospital, London, UK

**Keywords:** Gene therapy, potassium channels, molecular genetics, *KCNV2*, ERG, cone dystrophy, cone-rod dystrophy, retinal dystrophy, supernormal rod responses

## Abstract

-associated retinopathy or “cone dystrophy with supernormal rod responses” is an autosomal recessive cone-rod dystrophy with pathognomonic ERG findings. This gene encodes Kv8.2, a voltage-gated potassium channel subunit that acts as a modulator by shifting the activation range of the K^+^ channels in photoreceptor inner segments. Currently, no treatment is available for the condition. However, there is a lack of prospective long-term data in large molecularly confirmed cohorts, which is a prerequisite for accurate patient counselling/prognostication, to identify an optimal window for intervention and outcome measures, and ultimately to design future therapy trials. Herein we provide a detailed review of the clinical features, retinal imaging, electrophysiology and psychophysical studies, molecular genetics, and briefly discuss future prospects for therapy trials.

## Introduction

*KCNV2*-associated retinopathy (OMIM #610356) is an unusual, autosomal recessive cone-rod dystrophy with pathognomonic electroretinogram (ERG) findings (1–4). It was first described by Gouras et al in 1983 as a cone dystrophy with nyctalopia and supernormal rod responses (5). In the USA, it has an estimated frequency of 1/865,000 inhabitants and an incidence of 5 new cases per year (6). Wu et al. linked “cone dystrophy with supernormal rod responses” (CDSRR) to a 1.5 Mb region on chromosome 9p24, and subsequently identified disease-causing sequence variants in the *KCNV2* gene (7,8). *KCNV2* encodes a voltage-gated potassium channel, which sets vertebrate photoreceptor resting potential and voltage response (9).

This article aims to provide a detailed overview of the current clinical literature regarding *KCNV2* retinopathy, review our current understanding of the molecular genetics, and discuss potential novel treatments.

## Clinical presentation

Patients often present in the first or second decades of life with central scotoma, poor visual acuity, variable photophobia, and red-green axis dyschromatopsia with relative tritan sparing (3,4,10,11). Younger children may display an abnormal head posture, head shaking, and/or nystagmus, which can improve over time (12). Nyctalopia may also be reported at presentation, and patients often have mild to moderate myopia (13). A significant proportion of patients report both notable night blindness and photophobia, a combination of symptoms that is unusual in the early stages of a cone-rod dystrophy.

## Retinal imaging

**Figure 1.**
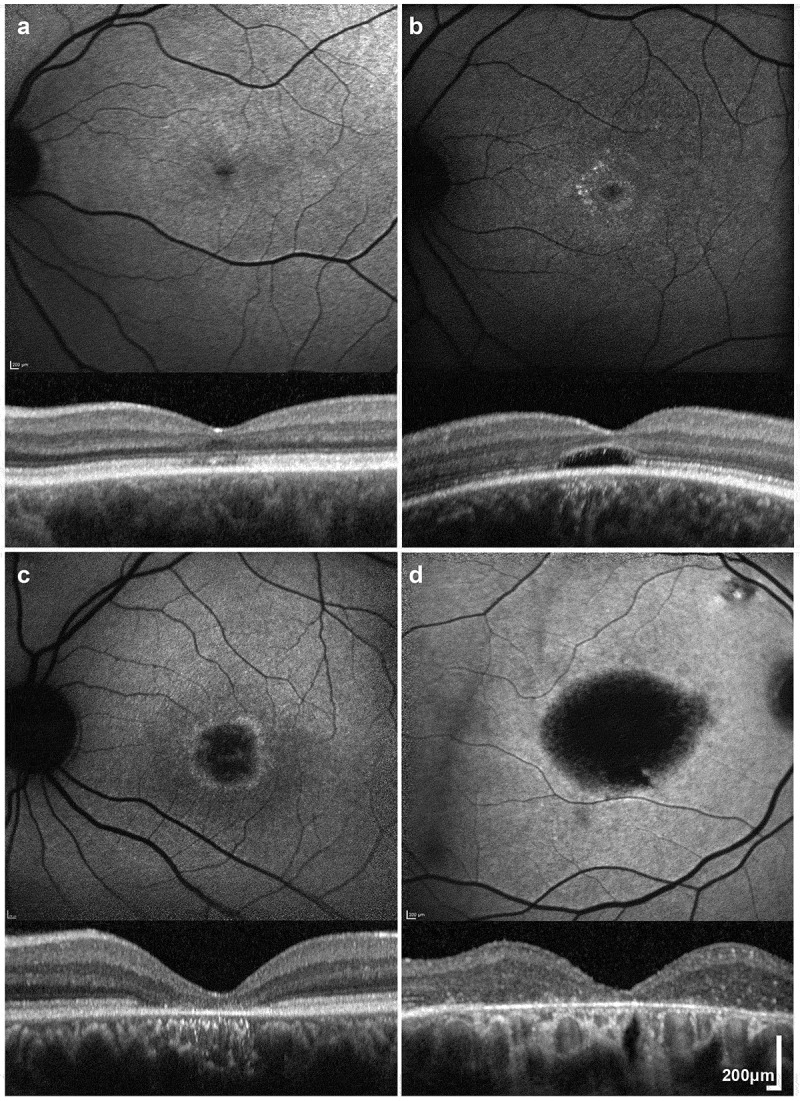
Retinal Imaging in *KCNV2-*Retinopathy. (a-d) Fundus autofluorescence (FAF) imaging with corresponding horizontal trans-foveal optical coherence tomography (OCT) scans of four patients with disease-causing *KCNV2* variants (a, b, c and d; 49, 25, 28 and 71 years of age respectively). A wide range of FAF patterns is observed: increased foveal signal (a), bull’s-eye maculopathy (b), perifoveal ring of increased signal with central atrophy (c and d). Corresponding OCT images show: small discontinuities and attenuation of the foveal ellipsoid zone (EZ) (a), a hyporeflective zone (b), and more extensive loss of the EZ and retinal pigment epithelial atrophy (c-d).

Fundus examination often reveals a relatively normal retinal periphery and a range of macular abnormalities, which vary from discrete accentuation of the foveal reflex to more pronounced macular retinal pigment epithelial (RPE) atrophy (10,13). Fundus autofluorescence (FAF) imaging reveals a wide range of findings including ring-like or bull’s-eye changes, increased foveal AF, and reduced central signal in keeping with atrophy, have all been reported (Figure 1). A parafoveal ring of increased AF is a common finding in younger patients, which may initially involve a broader area or multiple foci forming a concentric pattern in the second decade of life, and ultimately evolves into concentric areas of decreased signal indicative of RPE/photoreceptor dysfunction/loss (3,4,13).

Optical coherence tomography (OCT) identifies variable outer retinal integrity, from mild discontinuous reflectivity to more extensive loss of the ellipsoid zone (EZ), including a central hyporeflective zone (HRZ) in some patients (Figure 1) (4,14). Although foveal EZ changes are evident even in the earliest stages of the disease, there appears to be a relatively wide temporal window before significant atrophy is evident (14).

Adaptive optics scanning light ophthalmoscopy (AOSLO) is a non-invasive imaging modality that enables the visualization of photoreceptors at a microscopic level by correcting for ocular aberrations (15). AOSLO in *KCNV2* retinopathy reveals cone photoreceptor mosaic disruption with patches of absent and non-waveguiding cones and overall reduced cone density, but significant residual photoreceptors that could be therapeutically targeted (4).

## Electrophysiology, pupillometry and psychophysics

**Figure 2.**
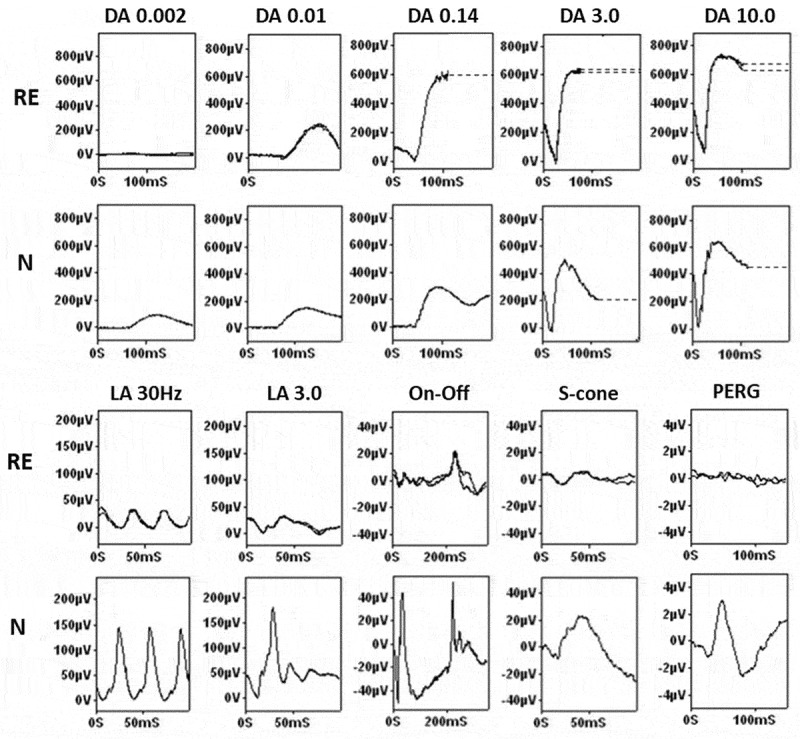
Electroretinography in *KCNV2*-Retinopathy. Full-field ERGs and PERG recorded from a patient with *KCNV2*-retinopathy (right eye; RE), compared with representative control recordings from an unaffected subject (N). Dark-adapted (DA) responses are shown for flash strengths of between 0.002 and 10.0 cd.s.m^−2^ (DA 0.002 – DA 10.0). In the case of *KCNV2*-retinopathy the DA 0.002 ERG is undetectable; DA 0.01 ERG is delayed and subnormal; the DA 10.0 ERG a-wave trough has a relatively broad shape of mildly subnormal amplitude with a late negative component; the DA 10 ERG b-wave is of relatively high amplitude. Light-adapted (LA) ERGs are shown for a flash strength 3.0 cd.s.m^−2^ (LA 30 Hz and 2 Hz); responses are reduced and delayed. Photopic On-Off ERGs show delay and reduction affecting both On and Off responses and S-cone ERGs are subnormal. The PERG P50 component is undetectable. Recordings were symmetrical and are shown for the right eye only. Abnormal traces are superimposed to demonstrate reproducibility with exception of DA 0.14 (single trace recorded). Broken lines replace blink artefacts that occur after the b-waves.

*KCNV2*-retinopathy has a pathognomonic ERG signature (Figure 2) (13,16). International Society for Clinical Electrophysiology of Vision (ISCEV) – standard (17) light-adapted (LA) ERGs are reduced and delayed, and photopic On-Off ERGs (18) typically show abnormalities of both cone-mediated On- and Off- systems (13). Under dark-adapted (DA) conditions, the rod-mediated dim flash (DA0.01) ERG is severely delayed and typically of subnormal amplitude; whereas to a strong flash the (DA10.0) ERG a-wave has a characteristic flattened trough of normal or near-normal amplitude with a late negative component, and the b-wave is of relatively high amplitude (and may be supernormal). Although not needed for diagnosis, a stimulus-response series reveals no detectable response to a very dim white flash (e.g. 0.002 cd.sm^−2^; detectable in healthy subjects), but there is a disproportionate increase in the ERG b-wave with increasing intermediate flash strengths. Pattern ERG P50 (19) is invariably undetectable, irrespective of age or fundus appearance, in keeping with severe macular dysfunction (13). In the largest cross-sectional study to date (n = 24), the ERG findings did not correlate with age, which suggests that the progressive structural macular degeneration can occur in the presence of relatively stable peripheral retinal function (13).

In 2019, Collison et al. performed pupillometry in two unrelated patients with molecularly confirmed *KCNV2-*retinopathy. They detected pupillary responses to moderate to high-luminance stimuli, including responses to high-luminance short-wavelength stimuli that were within normal limits. The normal sustained pupillary responses suggest an outer retinal locus and are consistent with ERG evidence of relatively preserved inner retinal function (20).

A detailed psychophysical investigation of 5 patients with *KCNV2*-retinopathy, concluded that the defect in the voltage-gated potassium channel produces a nonlinear distortion of the photoreceptor response after otherwise normal phototransduction (21). The authors thereby suggested that the previous name of the disorder (cone dystrophy with ‘supernormal’ rod ERG) to be potentially misleading, given their identification of comparable loss of both cone and rod photoreceptor function; also consistent with the mildly reduced DA10.0 ERG a-waves seen in most cases (13).

The combination of clinical, imaging, and ERG findings that characterize the phenotype of *KCNV2-*retinopathy are highly suggestive of the disease. Nevertheless, it remains possible that the condition is underdiagnosed due to a lack of clinical awareness of this particular phenotype, limited access to specialist ERG testing or failure to recognize the pathognomonic ERG features, which are not always associated with a DA strong flash ERG b-wave of abnormally high amplitude, and also a lack of genetic testing (13,16,22,23).

## Molecular genetics

*KCNV2* is a 2-exon gene, encoding a 545 amino acid protein, that was first cloned in 2002 (8). It is predominantly expressed in the heart and retina (24). When first described, the protein product was named Kv11.1, rather than Kv8.2, as it is known now; with the nomenclature change being that Kv11.1 was reassigned to a pore-forming subunit of a rapidly activating-delayed rectifier K^+^ channel, a product of the *KCNH2* gene (OMIM #152427). Kv8.2 is a regulatory subunit, which is known to be an electrically “silent” K^+^ channel subunit when expressed as a homotetramer. Initially, Ottschytsch et al. suggested that it combines with other proteins in heterotetrameric complexes. Indeed, Kv2.1 was found to generate current and promote trafficking of Kv6.3, Kv10.1 and Kv8.2, which supported his hypothesis (8). Through obligatory heteromerization with Kv2.1, Kv8.2 affects cellular excitability potential and alters the K^+^ current.

Four years later (2006), Wu et al. linked CDSRR to a 1.5 Mb region on chromosome 9p24 in a large multiply consanguineous family from UAE, and identified a homozygous nonsense variant in *KCNV2. In situ* hybridization using a *KCNV2* antisense riboprobe demonstrated its expression in the inner segments of human rod and cone photoreceptors (PR) (7). The importance of this gene in the visual cycle was further supported by Czirják et al., when it was suggested that the Kv2.1/Kv8.2 complex contributed to photoreception, which further explains why variants in *KCNV2* lead to a visual disorder (24). More recently, it has been proposed that the presence of Kv8.2 in the heteromeric complex regulates the function of the Kv2.1/Kv8.2 complex by shifting the activation range of the K^+^ channels in photoreceptor inner segments. Otherwise, as in the case of a dysfunctional *KCNV2* gene, the absence/reduced function of the subunit Kv8.2 in the potassium channels, would shift and depolarize the resting potential of the cells, which might account for the pathognomonic ERG findings in *KCNV2*-retinopathy (9).

In preparations of micro-dissected retinal neurons, the transcript levels of Kv8.2 and Kv2.1 were found to display daily rhythms, with elevated values during the night. It has been proposed that the transcriptional regulation of Kv8.2 and Kv2.1 is a mechanism by which the ‘retinal clock’ drives visual function according to different environmental lighting conditions (25).

Using chromatin immunoprecipitation and bioinformatic prediction analysis, two cone-rod homeobox (CRX) binding sites and one NRL binding site have been identified in the *KCNV2* promoter. Interestingly, shRNA-mediated knockdown of CRX binding sites in mouse models, resulted in reduced *KCNV2* promoter activity and low endogenous *KCNV2* mRNA expression in the retina, suggesting that retina-specific expression of *KCNV2* is controlled by the transcription factor *CRX* (26). These findings may be helpful in designing future gene therapy for *KCNV2-*retinopathy.

## Protein structure

**Figure 3.**
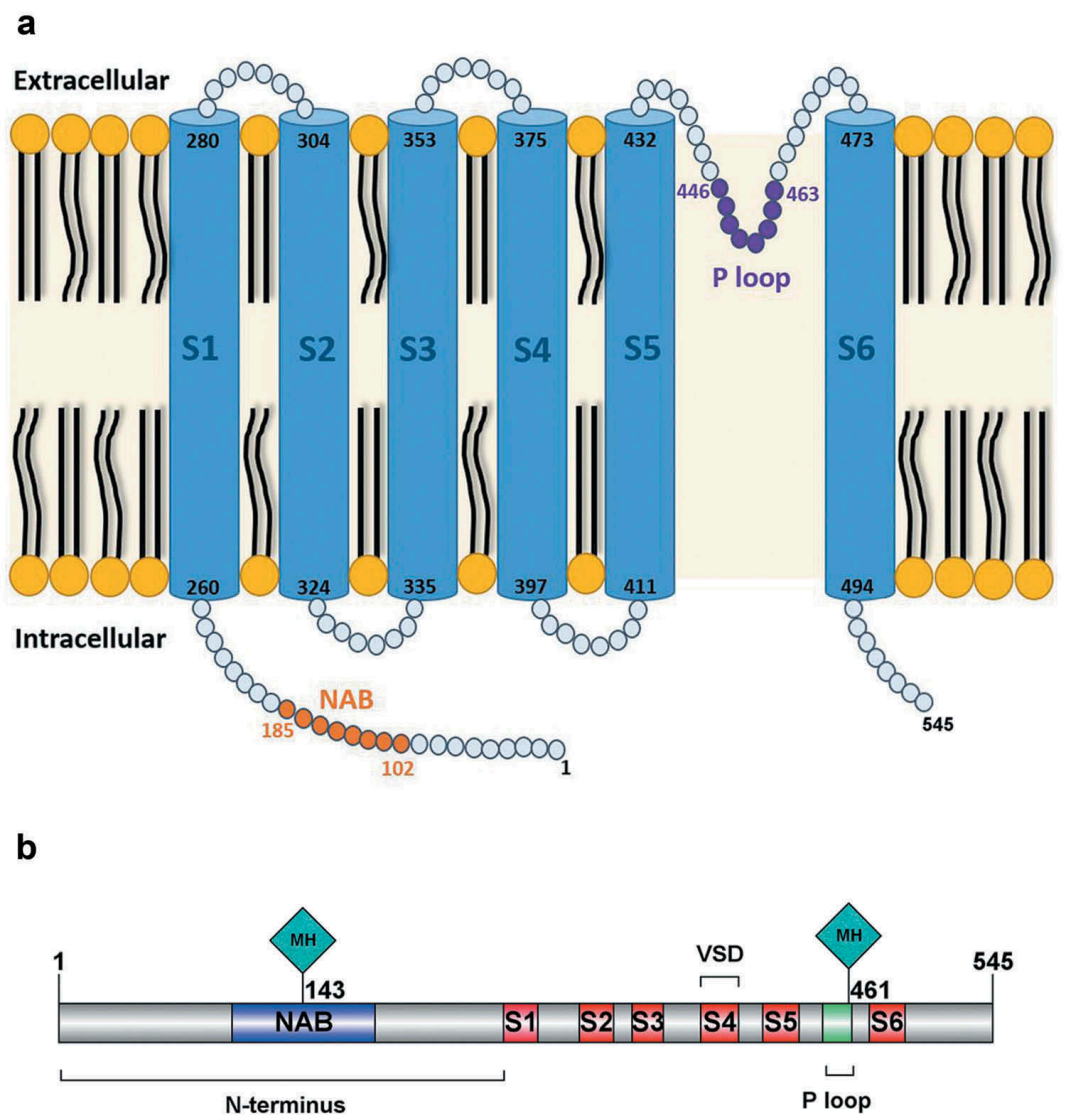
KCNV2 Protein Structure. (a) Graphical representation of the alpha-subunit of the potassium channel (Kv8.2) encoded by *KCNV2*. The subunit consists of: (i) a highly conserved tetramerization domain; N-terminal A and B box (NAB) that facilitates interaction between compatible alpha-subunits; (ii) 6 transmembrane domains (S1-S6); (iii) extracellular and intracellular loop segments; and (iv) an ultra-conserved potassium selective motif in the pore-forming loop between S5-S6 (P loop). (b) The two most frequently reported variants in patients with *KCNV2-*retinopathy are c.1381 G > A (p.Gly461Arg) and c.427 G > T (p.Glu143X), and may thereby represent mutational hotspots (MH); both locations are represented with a lozenge-shaped site in the gene annotation.

Voltage-dependent K^+^ channels are composed of alpha-subunits, which determine the structure of the channel, and beta-subunits which modulate its properties (27). *KCNV2* encodes Kv8.2, which is an alpha-subunit (8). Each channel subunit consists of: (i) an N-terminus with a highly conserved tetramerization domain known as N-terminal A and B box (NAB or T1) that facilitates interaction between compatible alpha-subunits; (ii) 6 transmembrane domains (S1-S6) with a positively-charged S4 that forms the voltage sensor domain (VSD); (iii) extracellular and intracellular loop segments; and (iv) an ultra-conserved potassium selective motif (Gly-Tyr-Gly) in the pore forming loop between S5-S6 (P loop), which forms the selective filter (28–33). A graphical representation of the protein and its domains is presented in Figure 3. Variants located in the intracellular amino-terminal region (T1) of Kv8.2 are very likely to be pathogenic as this domain lends stability to the channel structure. Variants in this region have produced elevated levels of non-functional monomers in a yeast model that were then degraded (34). However, using a different yeast model, it has been shown that sequence variants in T1 do not result in misfolding or fast degradation of the protein, but robustly prevent and disrupt interaction between the T1 domains of Kv8.2 and Kv2.1 (35).

## Reported sequence variants

More than 100 patients and 95 different variants have been reported across 22 studies (4,7,10–14,20-23,36–46). Supplementary table summarizes the previously described variants, including conservation, *in silico* prediction and frequency assessment. Of these, 46 are missense variants (two of which are located in the last codon and generate an extension of 61 amino acids), 21 nonsense variants, 14 intragenic deletions (13 causing a frameshift), 3 out-of-frame insertions (with subsequent frameshift), 4 duplications (2 causing frameshift), 6 gross deletions of an entire exon or the whole gene, and 1 complex rearrangement (c.19_1356 + 9571 delinsCATTTG; p.Arg7HisfsX57). Approximately two thirds of these variants are located in the amino-terminal region (N-terminus and NAB domains).

The most frequently reported variant is c.1381 G > A (p.Gly461Arg), located in the third residue of the ultra-conserved GYG-tripeptide motif (47). It has been reported as a disease-causing variant in 35 patients, either in the homozygous or compound heterozygous state (4,10,14,23,36–40). Moreover, it accounts for approximately 83% of all disease-causing variants reported in the P loop domain. Based on its frequency, p.Gly461Arg may represent a mutational hotspot (Figure 3). Another possible mutational hotspot is located in the amino-terminal A and B box (NAB), c.427 G > T (p.Glu143X). This nonsense variant causes premature protein termination and is predicted to cause loss of function. It has been reported in 31 patients (7,12,14) and accounts for approximately 41% of all disease-causing variants in the highly conserved NAB domain.

## Directions for therapy

There is currently no approved treatment for *KCNV2*-retinopathy, apart from symptomatic supportive measures including tinted spectacles/contact lenses and access to low visual aids/assistive technologies.

Gene supplementation therapy offers the possibility to improve the outcomes of several forms of monogenic inherited retinal disorders (IRD). It aims to deliver a “normal” copy of a defective gene that is no longer able to produce viable protein. Data from long-term follow-up studies of the pivotal gene therapy trials for *RPE65*-related retinal dystrophy (*RPE65*-RD) (OMIM #204100) are promising (48–50) and have resulted in the first FDA/EMA approved gene therapy for an ocular condition. One of the main factors that prompted interest in using gene therapy for *RPE65* was that despite the profound visual loss in animal models and humans, there was a wide window of *structural* photoreceptor preservation for therapeutic intervention (51).

*KCNV2*-retinopathy may also be a suitable target for gene therapy. Firstly, *KCNV2* is a small gene that can readily be packaged within the viral vector of choice, AAV. Secondly, there is a mouse model that recapitulates human disease very closely (including the ERG phenotype) and so can be targeted for therapeutic intervention (52). Thirdly, there are favourable functional and structural phenotypic features. In 1984, Alexander and Fishman reported three cases, of which two had the ‘typical’ supernormal rod ERGs but without nyctalopia, suggestive of good rod function despite abnormal scotopic ERG (53). Further functional studies have suggested that inner-retinal function and the phototransduction cascade are relatively normal in *KCNV2*-retinopathy (16,20). Structurally, although morphological changes at the fovea are evident on OCT in early stages of the disease, there appears to be a broad window of opportunity for therapeutic intervention before advanced structural changes and marked photoreceptor cell loss have occurred (13). This is supported by findings in the *KCNV2* knock-out mouse, where approximately 80% of cones are still intact by six months of age as compared to wild type, which if similar to humans, may allow for relatively late photoreceptor-directed treatment (52). However, further clinical and pre-clinical research, including prospective natural history studies, are needed to establish the optimal window for intervention, appropriate structural and functional (both retinal and visual) end-points to monitor both safety and efficacy, and identify participants most likely to benefit.

There are three main routes being explored to deliver a gene therapy product to the retina: via (i) intravitreal, (ii) subretinal or (iii) suprachoroidal injection. Although intravitreal injections are less invasive than subretinal injections and may be readily delivered by non-specialist surgeons, most currently available AAVs are unable to efficiently and reproducibly reach the outer retina – mainly due to the inner limiting membrane (ILM) acting as a physical barrier, thereby limiting transduction to the inner retinal layers (54). Modified AAVs – particularly serotype 2 – are proposed to be more effective in penetrating the ILM and allow broader transduction (55–59). However, in the case of *KCNV2-*retinopathy, which primarily affects photoreceptors, subretinal delivery of a gene therapy product is currently likely to be the most effective approach.

Pharmacological approaches with potassium channel modulators may provide a promising option for the treatment of several conditions, including cardiac arrhythmias, epilepsy, depression, autoimmune diseases and many others (60–63). Chemical agents that affect potassium channel functions may either activate or block current flow or alter channel gating (61). In theory, certain patients with *KCNV2-*retinopathy (depending on the effect of specific sequence variants on protein/channel structure/function) may also benefit from potassium channel modulators. How these might be safely delivered long term would also need to be addressed.

## Conclusions and future directions

Evidence from animal models and clinical studies identify *KCNV2-*retinopathy as a severe early onset retinal dystrophy with slowly progressive maculopathy, that might be amenable to future treatments. Phenotypic studies suggest that there is indeed relative structural preservation of retinal architecture and intact phototransduction (16,20,21,52). Multiple gene therapy trials for IRDs are ongoing (48,64–67), with the first approved gene therapy for *RPE65-*RD now available (NCT00999609). Further pre-clinical work in animal models and iPSC-derived models is needed to explore safety, efficacy and dosing of potential gene or drug therapy to facilitate translation to human clinical trials.

In the UK, *KCNV2* retinopathy accounts for 0.7% of the pedigrees with IRDs (68). Prospective data in large molecularly confirmed cohorts are the cornerstone for understanding the natural history of the disease. This is a prerequisite for the best-informed design of future therapy trials, as well as for patient counselling and advice on prognosis. Detailed phenotyping of patients with *KCNV2*-retinopathy will facilitate the identification of an optimal window for intervention, provide specific parameters to quantify treatment effects and define clinical endpoints, and help identify suitable patients for therapeutic intervention.

## Supplementary Material

Supplementary Material
