# Supplementary material for: KCNV2 retinopathy: clinical features, molecular genetics and directions for future therapy
Source: Ophthalmic Genet. 2020 May 22;41(3):208–15. doi: 10.1080/13816810.2020.1766087 (PMC7446039; doi:10.1080/13816810.2020.1766087)
Supplement: Supplementary Material [file IOPG_A_1766087_SM2520.pdf]

Supplementary Table: Previously reported variants associated with *KCNV2* retinopathy

Variant c. (p.)	Protein domain	Conservation (GERP score)	*In silico* prediction tools				Frequency in gnomAD	Reported in
			Provean	SIFT	Polyphen2	Grantham score		
c.6_9delCAAA (p.Lys3ArgfsX96)	N-terminus	-	-	-	-	-	14/282668 (0.000049)	[4]
c.7A>T (p.Lys3X)	N-terminus	-	-	-	-	-	Not reported	[3], [18]
c.8_11delAACA (p.Lys3fsX9)	N-terminus	-	-	-	-	-	Not reported	[15], [18], [20], [22]
c.19_1356+9571 delinsCATTTG (p.Arg7HisfsX57)	N-terminus	-	-	-	-	-	Not reported	[15], [20]
c.80G>A (p.Arg27His)	N-terminus	4.02	-0.78 (neutral)	0.01 (damaging)	Probably damaging 0.996 (sensitivity 0.55 and specificity 0.98)	29	375/282786 (0.00133)	[5]
c.107G>A (p.Arg36His)	N-terminus	1.12	-0.50 (neutral)	0.016 (damaging)	Benign 0.007 (sensitivity 0.96 and specificity 0.75)	29	19/282754 (0.000067)	[15]
c.137G>A (p.Trp46X)	N-terminus	-	-	-	-	-	Not reported	[16]
c.159C>G (p.Tyr53X)	N-terminus	-	-	-	-	-	Not reported	[6]
c.190G>A (p.Glu64Lys)	N-terminus	2.11	-0.34 (neutral)	0.076 (tolerated)	Benign 0.164 (sensitivity 0.92 and specificity 0.87)	56	4/251314 (0.000016)	[15]
c.200G>A (p.Trp67X)	N-terminus	-	-	-	-	-	1/251288 (0.000004)	[14]
c.217G>T (p.Glu73X)	N-terminus	-	-	-	-	-	1/31362 (0.000032)	[10]
c.222G>C (p.Glu74Asp)	N-terminus	1.05	-0.81 (neutral)	0.226 (tolerated)	Benign (0.015)	45	14/282668 (0.0000495)	[15]
c.226C>T (p.Gln76X)	N-terminus	-	-	-	-	-	Not reported	[10]
c.238G>T (p.Glu80X)	N-terminus	-	-	-	-	-	Not reported	[13]
c.240G>T (p.Glu80Asp)	N-terminus	-3.64	-0.63 (neutral)	0.163 (tolerated)	Benign (0.03)	45	Not reported	[6]
c.263G>A (p.Gly88Asp)	N-terminus	-1.0	-0.46 (neutral)	0.749 (tolerated)	Benign (0)	94	Not reported	[4], [20]
c.323_329del7 (p.Tyr108fsX14)	Amino terminal NAB	-	-	-	-	-	Not reported	[4] and [15]
c.325C>T (p.Gln109X)	Amino terminal NAB	-	-	-	-	-	3/231154 (0.000013)	[3]
c.328C>G (p.Leu110Val)	Amino terminal NAB	4.45	0.82 (neutral)	1.0 (tolerated)	Probably damaging (0.962)	32	Not reported	[15]
c.339C>A (p.Cys113X)	Amino terminal NAB	-	-	-	-	-	3/251064 (0.000012)	[4], [20]
c.357_358insC (p.120fsX371)	Amino terminal NAB	-	-	-	-	-	Not reported	[10]
c.377T>A (p.Leu126Gln)	Amino terminal NAB	3.77	-5.56 (deleterious)	0 (damaging)	Probably damaging 1.0 (sensitivity 0 and specificity 1.0)	113	Not reported	[3]
c.411_414delCCTG (p.Leu138fsX70)	Amino terminal NAB	-	-	-	-	-	Not reported	[12]
c.427G>T (p.Glu143X)	Amino terminal NAB	-	-	-	-	-	Not reported	[3], [6] and [11]
c.430C>T (p.Gln145X)	Amino terminal NAB	-	-	-	-	-	1/247098 (0.000004)	[3]
c.434_ 30+154del (p.Glu145LeufsX4)	Amino terminal NAB	-	-	-	-	-	Not reported	[15]
c.441C>G (p.Asp147Glu)	Amino terminal NAB	-2.81	-1.45 (neutral)	0.032 (damaging)	Probably damaging 0.998 (sensitivity 0.27 and specificity 0.99)	45	Not reported	[15]
c.442G>T (p.Glu148X)	Amino terminal NAB	-	-	-	-	-	3/247458 (0.000012)	[2], [10], [15], [20]
c.451T>C (p.Phe151Leu)	Amino terminal NAB	5.07	-5.58 (deleterious)	0 (damaging)	Probably damaging 1.0 (sensitivity 0 and specificity 1.0)	22	2/247528 (0.000008)	[11]
c.451T>G (p.Phe151Val)	Amino terminal NAB	5.07	-6.51 (deleterious)	0 (damaging)	Probably damaging 1.0 (sensitivity 0 and specificity 1.0)		Not reported	[17]
c.454G>A (p.Asp152Asn)	Amino terminal NAB	5.07	-4.82 (deleterious)	0 (damaging)	Probably damaging 1.0 (sensitivity 0 and specificity 1.0)	23	1/247512 (0.000004)	[8]
c.460_461insCG (p.Asp154fsX58)	Amino terminal NAB	-	-	-	-	-	Not reported	[13]
c.473T>G (p.Phe158Cys)	Amino terminal NAB	4.98	-7.79 (deleterious)	0 (damaging)	Probably damaging 1.0 (sensitivity 0 and specificity 1.0)	205	Not reported	[1]
c.491T>C (p.Phe164Ser)	Amino terminal NAB	4.98	-7.66 (deleterious)	0 (damaging)	Probably damaging 1.0 (sensitivity 0 and specificity 1.0)	155	1/248040 (0.000004)	[15]
c.520dupG (p.Asp174fsX198)	Amino terminal NAB	-	-	-	-	-	Not reported	[14]
c.529T>C (p.Cys177Arg)	Amino terminal NAB	4.91	-11.38 (deleterious)	0 (damaging)	Probably damaging 1.0 (sensitivity 0 and specificity 1.0)	180	1/244508 (0.0000041)	[5] and [8]
c.531T>A (p.Cys177X)	Amino terminal NAB	-	-	-	-	-	1/244106 (0.0000041)	[16]
c.533C>T (p.Pro178Leu)	Amino terminal NAB	4.91	-4.38 (deleterious)	0.168 (tolerated)	Probably damaging 1.0 (sensitivity 0 and specificity 1.0)	98	4/243626 (0.000016)	[1]
c.550G>A (p.Glu184Lys)	Amino terminal NAB	4.91	-3.75 (deleterious)	0.001 (damaging)	Probably damaging 1.0 (sensitivity 0 and specificity 1.0)	56	Not reported	[10]
c.551A>T (p.Glu184Val)	Amino terminal NAB	4.91	-6.58 (deleterious)	0 (damaging)	Probably damaging 1.0 (sensitivity 0 and specificity 1.0)	121	Not reported	[10]
c.556_571del16 (p.Gly186fsX96)	N-terminus	-	-	-	-	-	Not reported	[4]

Supplementary Table: Previously reported variants associated with *KCNV2* retinopathy

Variant c. (p.)	Protein domain	Conservation (GERP score)	*In silico* prediction tools				Frequency in gnomAD	Reported in
			Provean	SIFT	Polyphen2	Grantham score		
c.563G>A (p.Trp188X)	N-terminus	-	-	-	-	-	1/236686 (0.0000042)	[4]
c.564G>C (p.Trp188Cys)	N-terminus	4.91	-12.33 (deleterious)	0 (damaging)	Probably damaging 1.0 (sensitivity 0 and specificity 1.0)	215	2/236686 (0.0000084)	[3]
c.568delG (p.Gly189fsX21)	N-terminus	-	-	-	-	-	Not reported	[11]
c.592T>A (p.Cys198Ser)	N-terminus	4.91	-9.48 (deleterious)	0 (damaging)	Probably damaging 1.0 (sensitivity 0 and specificity 1.0)	112	1/224114 (0.0000045)	[1]
c.617G>C (p.Arg206Pro)	N-terminus	4.80	-4.09 (deleterious)	0.001 (damaging)	Probably damaging 1.0 (sensitivity 0 and specificity 1.0)	103	Not reported	[5]
c.638G>C (p.Arg213Pro)	N-terminus	3.72	-1.50 (neutral)	0.008 (damaging)	Benign 0.041	103	24/249186 (0.000096)	[1]
c.647T>C (p.Ile216Thr)	N-terminus	4.87	-0.78 (neutral)	0.571 (tolerated)	Possibly damaging 1.0 (sensitivity 0 and specificity 1.0)	89	271/250532 (0.001082)	[4]
c.655G > T (Glu219X)	N-terminus	-	-	-	-	-	2/218764 (0.0000091)	[22]
c.667C>T (p.Gln223X)	N-terminus	-	-	-	-	-	Not reported	[13]
c.721C>T and c.722C>A (p.Pro241X)	N-terminus	-	-	-	-	-	Multi-nucleotide variants; freq of 13/261268 (0.00005) and 2/260850 (0.0000077)	[1]
c.725A>G (p.Gln242Arg)	N-terminus	0.65	-0.01 (neutral)	0.348 (tolerated)	Benign 0.003	43	1/31368 (0.000032)	[15]
c.727C>T (p.Arg243Trp)	N-terminus	4.84	-6.85 (deleterious)	0 (damaging)	Probably damaging 1.0 (sensitivity 0 and specificity 1.0)	101	2/229634 (0.000009)	[15]
c.758delC (p.Pro253fsX68)	N-terminus	-	-	-	-	-	Not reported	[12]
c.767C>G (p.Ser256Trp)	N-terminus	4.94	-6.33 (deleterious)	0 (damaging)	Probably damaging 1.0 (sensitivity 0 and specificity 1.0)	177	Not reported	[3]
c.776C>T (p.Ala259Val)	N-terminus	4.94	-3.92 (deleterious)	0.001 (damaging)	Probably damaging 0.994 (sensitivity 0.69 and specificity 0.97)	64	Not reported	[3]
c.775_795dup21 (p.Ala259_Ala265dup7)	N-terminus/S1	-	-	-	-	-	Not reported	[15]
c.778A>T (p.Lys260X)	S1	4.94	-	-	-	-	9/269892 (0.000033)	[1], [10] and [15]
c.782C>A (p.Ala261Asp)	S1	0.21	-2.85 (deleterious)	0.003 (damaging)	Possibly damaging 0.756 (sensitivity 0.85 and specificity 0.92)	126	2/238640 (0.000008)	[4], [12] and [15]
c.794_795dupCC (p.Ser266fsX57)	S1	-	-	-	-	-	Not reported	[15]
c.853A>T (p.Met285Leu)	EC1	3.92	0.33 (neutral)	0.97 (tolerated)	Benign (0.003)	15	Not reported	[15]
c.859C>T (p.Gln287X)	EC1	-	-	-	-	-	6/246422 (0.000024)	[10]
c.867delC (p.Ser289TrpfsX33)	EC1	-	-	-	-	-	2/246794 (0.000008)	1
c.874G>A (p.Gly292Ser)	EC1	4.98	-0.59 (neutral)	0.051 (tolerant)	Probably damaging 0.997 (sensitivity 0.41 and specificity 0.98)	56	6/247114 (0.000024)	[15]
c.887_888 delCA (Pro296delCA)	EC1	-	-	-	-	-	Not reported	[22]
c.916G>T (p.Glu306X)	S2	-	-	-	-	-	Not reported	[3], [18]
c.958C>T (p.Arg320Cys)	S2	5.22	-7.75 (deleterious)	0 (damaging)	Probably damaging 1.0 (sensitivity 0 and specificity 1.0)	180	8/281052 (0.000028)	[12]
c.964G>C (p.Ala322Pro)	S2	1.24	-2.03 (neutral)	0.31 (damaging)	Probably damaging 0.948 (sensitivity 0.79 and specificity 0.95)	27	Not reported	[11]
c.989T>C (p.Phe330Ser)	Intracellular loop between S2-S3	5.22	-7.66 (deleterious)	0 (damaging)	Probably damaging 1.0 (sensitivity 0 and specificity 1.0)	155	Not reported	[15]
c.996_997insGC (p.Ser333AlafsX121)	Intracellular loop between S2-S3	-	-	-	-	-	3/248978 (0.000012)	[12], [19]
c.1001delC (p.Ala334fsX453)	Intracellular loop between S2-S3	-	-	-	-	-	Not reported	[2], [10], [20]
c.1016_1024delACCTGGTGG (p.del Asp339_Val341)	S3	-	-	-	-	-	5/280066 (0.000018)	[3], [10], [13], [15], [17], [18]
c.1123G>A (p.Val375Met)	S4	5.07	-2.59 (deleterious)	0.018 (damaging)	Probably damaging 0.995 (sensitivity 0.68 and specificity 0.97)	21	109/278618 (0.00039)	[4]
c.1132G>A (p.Val378Ile)	S4	-4.24	0.61 (neutral)	1.0 (tolerated)	Benign 0.005 (sensitivity of 0.97 and specificity of 0.74)	29	3/278648 (0.00001)	[4]
c.1133_1141dup (p.Leu381_Arg383dup)	S4	-	-	-	-	-	29/278648 (0.0001)	[15]
c.1199delT (p.Phe400fsX53)	Intracellular loop between S4-S5	-	-	-	-	-	4/247732 (0.000016)	[11], [18]
c.1211T>C (p.Leu404Pro)	Intracellular loop between S4-S5	5.22	-6.42 (deleterious)	0 (damaging)	Probably damaging 0.999 (sensitivity 0.14 and specificity 0.99)	98	Not reported	[15], [20]

Supplementary Table: Previously reported variants associated with *KCNV2* retinopathy

Variant c. (p.)	Protein domain	Conservation (GERP score)	*In silico* prediction tools				Frequency in gnomAD	Reported in
			Provean	SIFT	Polyphen2	Grantham score		
c.1318C>T (p.Thr439Ile)	Extracellular between S5 and P loop	4.36	-3.85 (deleterious)	0.012 (damaging)	Probably damaging 0.997 (sensitivity 0.41 and specificity 0.98)	89	Not reported	[11]
c.1348T>A (p.Trp450Arg)	P loop	5.44	-13.52 (deleterious)	0 (damaging)	Probably damaging 1.0 (sensitivity 0 and specificity 1.0)	101	1/245454 (0.0000041)	[15]
c.1348T>G (p.Trp450Gly)	P loop	5.44	-12.56 (deleterious)	0 (damaging)	Probably damaging 0.998 (sensitivity 0.27 and specificity 0.99)	184	5/245454 (0.00002037)	[1]
c.1376G>A (p.Gly459Asp)	P loop	5.37	-6.58 (deleterious)	0.003 (damaging)	Probably damaging 1.0 (sensitivity 0 and specificity 1.0)	94	Not reported	[3]
c.1381G>A (p.Gly461Arg)	P loop	5.37	-7.53 (deleterious)	0.005 (damaging)	Probably damaging 1.0 (sensitivity 0 and specificity 1.0)	125	35/282736 (0.00012)	[1], [2], [4], [5], [7], [10], [11], [13] and [15], [18]
c.1381G>T (p.Gly461X)	P loop	-	-	-	-	-	6/282736 (0.000021)	[17]
c.1404delC (His468fsX503)	Extracellular between P loop and S6	-	-	-	-	-	[2]	[2]
c.1409G>A (p.Gly470Asp)	C-terminus	5.37	-6.66 (deleterious)	0 (damaging)	Probably damaging 1.0 (sensitivity 0 and specificity 1.0)		0	[9]
c.1607A>G (p.Asn536Ser)	C-terminus	2.80	-0.2 (neutral)	0.27 (tolerated)	Benign 0.001 (sensitivity 0.99 and specificity 0.15)	46	483/282678 (0.002)	[15]
c.1616T>C (p.Leu539Pro)	C-terminus	3.52	-0.59 (neutral)	1.0 (tolerated)	Benign 0 (sensitivity 1.0 and specificity 0)	98	1162/282646 (0.004)	[15]
c.1637T>C (p.X546GlnextX61)	C-terminus	-	-	-	-	-	Not reported	[1]
c.1638G>T (p.Ter546Tyr Ext61)	C-terminus	-	-	-	-	-	1/250810 (0.000004)	[17]
del of exon 2 (Val453_Asn545del)	-	-	-	-	-	-	Not reported	[17]
g.2570596_2807413del (complete gene del)	-	-	-	-	-	-	-	[15]
g.2570596_2807413del	-	-	-	-	-	-	-	[4]
g.2657638_2737340del (complete gene del)	-	-	-	-	-	-	-	[15], [20]
g.2696639_2713626del (del exon 1+)	-	-	-	-	-	-	-	[15]
92.7kb del of chr 9p24.2 from bp 2,673,984 to 2,766,722	-	-	-	-	-	-	-	[21]

1. Thiagalingam S, McGee TL, Weleber RG, Sandberg MA, Trzupek KM, Berson EL, Dryja TP. Novel mutations in the KCNV2 gene in patients with cone dystrophy and a supernormal rod electroretinogram. *Ophthalmic Genet*. 2007 Sep;28(3):135-42.
2. Ben Salah S, Kamei S, Sénéćhal A, Lopez S, Bazalgette C, Bazalgette C, Eliaou CM, Zanlonghi X, Hamel CP. Novel KCNV2 mutations in cone dystrophy with supernormal rod electroretinogram. *Am J Ophthalmol*. 2008 Jun;145(6):1099-106.
3. Wu H, Cowing JA, Michaelides M, Wilkie SE et al. Mutations in the gene KCNV2 encoding a voltage-gated potassium channel subunit cause "cone dystrophy with supernormal rod electroretinogram" in humans. *Am J Hum Genet*. 2006 Sep;79(3):574-9.
4. Thiadens AA, Phan TM, Zekveld-Vroon RC, Leroy BP, van den Born LI, Hoyng CB, Klaver CC; Writing Committee for the Cone Disorders Study Group Consortium, Roosing S, Pott JW, van Schooneveld MJ, van Moll-Ramirez N, van Genderen MM et al. Clinical course, genetic etiology, and visual outcome in cone and cone-rod dystrophy. *Ophthalmology*. 2012 Apr;119(4):819-26.
5. Fujinami K, Tsunoda K, Nakamura N, Kato Y, Noda T, Shinoda K, Tomita K, Hatase T, Usui T, Akahori M, Itabashi T et al. Molecular characteristics of four Japanese cases with KCNV2 retinopathy: report of novel disease-causing variants. *Mol Vis*. 2013 Jul 20;19:1580-90.
6. Khan AO, Alrashed M, Alkuraya FS. 'Cone dystrophy with supranormal rod response' in children. *Br J Ophthalmol*. 2012 Mar;96(3):422-6.
7. Friedburg C, Wissinger B, Schambeck M, Bonin M, Kohl S, Lorenz B. Long-term follow-up of the human phenotype in three siblings with cone dystrophy associated with a homozygous p.G461R mutation of KCNV2. *Invest Ophthalmol Vis Sci*. 2011 Nov 7;52(12):8621-9.
8. Oishi M, Oishi A, Gotoh N, Ogino K, Higasa K, Iida K, Makiyama Y, Morooka S, Matsuda F, Yoshimura N. Next-generation sequencing-based comprehensive molecular analysis of 43 Japanese patients with cone and cone-rod dystrophies. *Mol Vis*. 2016 Feb 20;22:150-60.
9. Huang L, Xiao X, Li S, Jia X, Wang P, Sun W, Xu Y, Xin W, Guo X, Zhang Q. Molecular genetics of cone-rod dystrophy in Chinese patients: New data from 61 probands and mutation overview of 163 probands. *Exp Eye Res*. 2016 May;146:252-8.
10. Wissinger B, Dangel S, Jägle H, Hansen L, Baumann B, Rudolph G et al. Cone dystrophy with supernormal rod response is strictly associated with mutations in KCNV2. *Invest Ophthalmol Vis Sci*. 2008 Feb;49(2):751-7.
11. Sergouniotis PI, Holder GE, Robson AG, Michaelides M, Webster AR, Moore AT. High-resolution optical coherence tomography imaging in KCNV2 retinopathy. *Br J Ophthalmol*. 2012 Feb;96(2):213-7.
12. Zelinger L, Wissinger B, Eli D, Kohl S, Sharon D, Banin E. Cone dystrophy with supernormal rod response: novel KCNV2 mutations in an underdiagnosed phenotype. *Ophthalmology*. 2013 Nov;120(11):2338-43.
13. Vincent A, Wright T, Garcia-Sanchez Y, Kisilak M, Campbell M, Westall C, Héon E. Phenotypic Characteristics Including In Vivo Cone Photoreceptor Mosaic in *KCNV2*-Related "Cone Dystrophy with Supernormal Rod Electroretinogram". *Invest Ophthalmol Vis Sci*. 2013 Jan 30;54(1):898-908.

14. Kutsuma T, Katagiri S, Hayashi T, Yoshitake K, Iejima D, Gekka T, Kohzaki K, Mizobuchi K, Baba Y, Terauchi R, Matsuura T et al. Novel biallelic loss-of-function KCNV2 variants in cone dystrophy with supernormal rod responses. *Doc Ophthalmol*. 2019 Jun;138(3):229-239.

15. Wissinger B, Schaich S, Baumann B, Bonin M, Jägle H, Friedburg C, Varsányi B, Hoyng CB, Dollfus H, Heckenlively JR, Rosenberg T, Rudolph G, Kellner U, Salati R, Plomp A et al. Large deletions of the KCNV2 gene are common in patients with cone dystrophy with supernormal rod response. *Hum Mutat*. 2011 Dec;32(12):1398-406.

16. Lenis TL, Dhrami-Gavazi E, Lee W, Mukkamala SK, Tabacaru MR, Yannuzzi L, Gouras P, Tsang SH. Novel compound heterozygous mutations resulting in cone dystrophy with supernormal rod response. *JAMA Ophthalmol*. 2013 Nov;131(11):1482-5.

17. Robson AG, Webster AR, Michaelides M, Downes SM, Cowing JA, Hunt DM, Moore AT, Holder GE. "Cone dystrophy with supernormal rod electroretinogram": a comprehensive genotype/phenotype study including fundus autofluorescence and extensive electrophysiology. *Retina*. 2010 Jan;30(1):51-62.

18. Stockman A, Henning GB, Michaelides M, Moore AT, Webster AR, Cammack J, Ripamonti C. Cone dystrophy with "supernormal" rod ERG: psychophysical testing shows comparable rod and cone temporal sensitivity losses with no gain in rod function. *Invest Ophthalmol Vis Sci*. 2014 Feb 10;55(2):832-40.

19. Xu D, Su D, Nusinowitz S, Sarraf D. Central ellipsoid loss associated with cone dystrophy and KCNV2 mutation. *Retin Cases Brief Rep*. 2018 Fall;12 Suppl 1:S59-S62.

20. Zobor D, Kohl S, Wissinger B, Zrenner E, Jägle H. Rod and cone function in patients with KCNV2 retinopathy. *PLoS One*. 2012;7(10):e46762.

21. Grigg JR, Holder GE, Billson FA, Korsakova M, Jamieson RV. The importance of electrophysiology in revealing a complete homozygous deletion of KCNV2. *J AAPOS*. 2013 Dec;17(6):641-3.

22. Collison FT, Park JC, Fishman GA, Stone EM, McAnany JJ. Two-color pupillometry in KCNV2 retinopathy. Doc Ophthalmol. 2019 Aug;139(1):11-20.
